# Phthiriasis palpebrarum with scalp and pubic hair infestation

**DOI:** 10.5935/0004-2749.2023-0213

**Published:** 2023-11-24

**Authors:** Yuanfeng Xue, Yuanyuan Zhang, Xiaoting Dai, Bangtao Yao

**Affiliations:** 1 Department of General Practice, Nanjing Lishui District Baima Health Hospital, Nanjing, Jiangsu Province, China; 2 Department of Neurosurgery, Nanjing Lishui People’s Hospital, Zhongda Hospital Lishui branch, Southeast University, Nanjing, Jiangsu Province, China; 3 Department of Rehabilitation, Nanjing Lishui People’s Hospital, Zhongda Hospital Lishui branch, Southeast University, Nanjing, Jiangsu Province, China; 4 Department of Infectious Diseases, Nanjing Lishui People’s Hospital, Zhongda Hospital Lishui branch, Southeast University, Nanjing, Jiangsu Province, China; 5 Department of Ophthalmology, Nanjing Lishui People’s Hospital, Zhongda Hospital Lishui branch, Southeast University, Nanjing, Jiangsu Province, China

Dear Editor,

*Phthirus pubis* are clawed bloodsucking insects^(1)^ whose infestation is considered a highly infectious tropical parasitic disease, nearly affecting 400 million of the human population worldwide. It is generally transmitted through sexual behaviors or close body contact^(1,2)^. Almost 30% of patients with *P. pubis* infestation are closely related to sexually transmitted diseases, including gonorrhea, human immunodeficiency virus (HIV), and syphilis^(1,3)^.

To our knowledge, *P. pubis* infestation of the eyelids and eyelashes (Phthiriasis palpebrarum) with scalp and pubic hair infestation is extremely rare. Moreover, phthiriasis palpebrarum is usually misdiagnosed as blepharoconjunctivitis and eyelid eczema^(2,4)^. Detecting lice and nits in the affected areas of the body aids to establish a definitive diagnosis. Slit lamp and dermatoscopic examinations were proven to be non-invasive and effective for diagnosing *P. pubis*infestation^(2,3)^.

**Figure 1:**
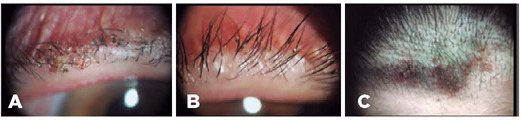
Slit lamp examination of both eyes indicated several adult lice with ingested blood and nits that adhered to the eyelids and eyelashes (A, B). Lice and nits were further found on the forehead (C).

A 70-year-old Chinese woman visited our clinical center presenting with a history of bilateral persistent irritation and itching of the eyelids for 1 month. Upon admission, her conjunctiva appeared reddish. The slit lamp examination of both eyes indicated that several adult lice with ingested blood and nits adhered to the eyelids and eyelashes (Figure 1A, B). After a detailed inspection, the lice and nits were further found on the forehead (Figure 1C) and pubic hairs. Eyebrows, chest, and axillary hairs were unremarkable. On light microscopy, the nits were observed to be firmly attached to the shaft of the eyelashes. The laboratory tests for HIV, syphilis, gonorrhea, and hepatitis B and C were conducted and showed negative results. The patient’s husband was advised for a detailed check-up and apparently lice and nits were also visible on his pubic area.

**Figure 2:**
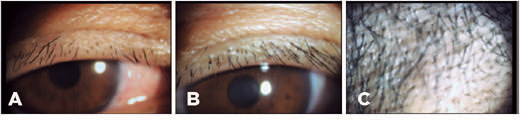
During a 1-month follow-up period, lice and nits were undetec-table on the eyelids, eyelashes (A, B), and forehead (C).

The patient was treated with the manual removal of lice and nits with fine forceps under an operating microscopy combined with involved hair trimming. A phenothrin shampoo was applied for the scalp and pubic infestation. During a two week follow-up period, her symptoms resolved completely, and lice and nits were undetectable on the eyelids, eyelashes (Figure 2A, B), and forehead (Figure 2C). No recurrence was observed during the 1 month follow-up visit. Moreover, she and her husband were also advised to be treated simultaneously. The clothing, bed linen, and towels of the family members were washed separately in hot water.

*Pubis* infestation is a highly infectious tropical parasitic disease and transmitted rapidly with a worldwide prevalence, in which nearly 400 million of the human population are affected^(1,2)^. It is generally transmitted through sexual behaviors or close physical touch. It mainly occurs in hair-bearing regions, such as the perineal region, axillae, and groin. It is also a predictor for poor hygiene and living environment^(2)^.

To our knowledge, phthiriasis palpebrarum with scalp and pubic hair infestation is extremely rare. The diagnosis of *P. pubis* infestation is made by finding insects and nits in the affected areas of the body. Slit lamp and dermatoscopic investigations were proven to be effective and helpful for diagnosing *P. pubis* infestation.^(2,3)^ Phthiriasis palpebrarum is generally misdiagnosed as eyelid eczema and blepharoconjunctivitis, since patients with *P. pubis* infestation often presents with pruritus, irritation, and conjunctival congestion^(3,4)^.

The treatment for *P. pubis* infestation involves the elimination of the parasites, which is considered as a challenging approach. Conventional treatments include medical and surgical options. The topical application of several pediculicides, such as 1% lindane lotion, phenothrin, and malathion, was proven to be an effective treatment method. However, these treatments were harmful to the eyes.^(3,5)^ Although petrolatum is useful for killing lice on the eyelashes and adjacent eyelids, it cannot destroy the nits^(5)^. Recent reports have documented that hair trimming of the affected areas, combined with the removal of lice and nits under an operating microscopy, is proven effective and safe^(3)^.

Further inspection for all hair-bearing areas of patients with *P. pubis* infestation needs to be conducted. In this study, almost 30% of patients were associated with sexually transmitted diseases.^(3)^ Therefore, all family members and sexual partners should be checked simultaneously in some cases due to the high risk of spread of *P. pubis* infestation. Thus, the clothing, bed linen, and towels of the family need to be washed separately in hot water.
